# Ultra-High-Precision, *in-vivo* Pharmacokinetic Measurements Highlight the Need for and a Route Toward More Highly Personalized Medicine

**DOI:** 10.3389/fmolb.2019.00069

**Published:** 2019-08-16

**Authors:** Philip A. Vieira, Christina B. Shin, Netzahualcóyotl Arroyo-Currás, Gabriel Ortega, Weiwei Li, Arturo A. Keller, Kevin W. Plaxco, Tod E. Kippin

**Affiliations:** ^1^Department of Psychology, California State University, Dominguez Hills, Carson, CA, United States; ^2^Institute for Collaborative Biotechnologies, University of California, Santa Barbara, Santa Barbara, CA, United States; ^3^Department of Psychological & Brain Sciences, University of California, Santa Barbara, Santa Barbara, CA, United States; ^4^Department of Pharmacology and Molecular Sciences, Johns Hopkins University School of Medicine, Baltimore, MD, United States; ^5^Department of Chemistry and Biochemistry, University of California, Santa Barbara, Santa Barbara, CA, United States; ^6^Center for Bioengineering, University of California, Santa Barbara, Santa Barbara, CA, United States; ^7^Bren School of Environmental Science & Management, University of California, Santa Barbara, Santa Barbara, CA, United States; ^8^Interdepartmental Program in Biomolecular Science and Engineering, University of California, Santa Barbara, Santa Barbara, CA, United States; ^9^Neuroscience Research Institute, University of California, Santa Barbara, Santa Barbara, CA, United States; ^10^Department of Molecular Cellular and Developmental Biology, University of California, Santa Barbara, Santa Barbara, CA, United States

**Keywords:** aptamer-based sensors, therapeutic drug monitoring, pharmacokinetics, aminoglycosides, body surface area

## Abstract

Clinical drug dosing would, ideally, be informed by high-precision, patient-specific data on drug metabolism. The direct determination of patient-specific drug pharmacokinetics (“peaks and troughs”), however, currently relies on cumbersome, laboratory-based approaches that require hours to days to return pharmacokinetic estimates based on only one or two plasma drug measurements. In response clinicians often base dosing on age, body mass, pharmacogenetic markers, or other indirect estimators of pharmacokinetics despite the relatively low accuracy of these approaches. Here, in contrast, we explore the use of indwelling electrochemical aptamer-based (E-AB) sensors as a means of measuring pharmacokinetics rapidly and with high precision using a rat animal model. Specifically, measuring the disposition kinetics of the drug tobramycin in Sprague-Dawley rats we demonstrate the *seconds* resolved, real-time measurement of plasma drug levels accompanied by measurement validation via HPLC-MS on *ex vivo* samples. The resultant data illustrate the significant pharmacokinetic variability of this drug even when dosing is adjusted using body weight or body surface area, two widely used pharmacokinetic predictors for this important class of antibiotics, highlighting the need for improved methods of determining its pharmacokinetics.

## Introduction

A drug's pharmacokinetics can vary widely from patient to patient (Kiang et al., 2014; Reichel and Lienau, 2016) due to, for example, genetic differences (Hayashi, 2013). Indeed, clinically significant variability is even seen day-to-day within a single individual (DeGorter et al., 2012; Schell et al., 2014) due to, for example, drug interactions (Zhang et al., 2009), diet (Bressler, 2006; El-Demerdash et al., 2012; Toh et al., 2014), physical activity (Sidhu et al., 2011; Janukonyte et al., 2014; Zourikian et al., 2016) or health status (Roberts and Lipman, 2009; Roberts et al., 2014; Sinnollareddy et al., 2015). To combat this variability clinicians are increasingly turning to personalized medicine, which seeks to tailor treatment to the individual, taking into consideration each patient's unique response to therapy, with the goal being to maximize efficacy, and minimize adverse reactions (Aspinall and Hamermesh, 2007).

Ideally, personalized medicine would be informed by high-precision, patient-specific data regarding drug pharmacokinetics. The accuracy with which current methods (“peaks and troughs”) are able to define this, however, is poor as the approach relies on only one or two measurements of plasma drug levels (Gross, 1998). Moreover, as the approach requires blood draws and laboratory measurements it is also slow and cumbersome, returning an answer only hours to days after sample collection (Gross, 1998). Because of these limitations dose determination is thus instead often performed using indirect predictors of pharmacokinetics (Poulin et al., 2011). Among these are the century-old body surface area (BSA)-based calculations, the first of which was derived from the data of only 9 patients (Shuter and Aslani, 2000) and went unconfirmed for 60 years (Haycock et al., 1978). Even today, BSA-based dose determination remains common, beginning in Phase I preclinical trials (FDA), where the BSA-pharmacokinetic relationships of animals are based on body mass and scaled non-linearly to infer the appropriate BSA-based dosing for humans (Reagan-Shaw et al., 2008; Nair and Jacob, 2016).

Despite its continued, widespread use, BSA-based dose determination suffers from potentially serious limitations. First, it is poorly correlated with the pharmacokinetics of many classes of drugs (Gurney, 1996; Dooley and Poole, 2000; Felici et al., 2002), often leading to suboptimal dosing (Sawyer and Ratain, 2001; Baker et al., 2002; Gao et al., 2008), including both inefficacious underdosing, and potentially lethal overdosing (Grochow et al., 1990). Second, BSA calculations fail to account for abnormal body sizes (Gurney and Shaw, 2007; Pai, 2012) or genetic variation across populations (Gurney, 1996, 2002). The field of pharmacogenetics seeks to overcome that latter factor by tailoring dosing to the specific genetic background of individual patients, but to date this is still poorly understood, lacks consistency in clinical outcomes, and has failed to adequately predict individual drug response (Shah and Shah, 2012). Simply put, current approaches to dose-determination fall far short of the demands of truly personalized, high-precision medicine.

**Figure 1:**
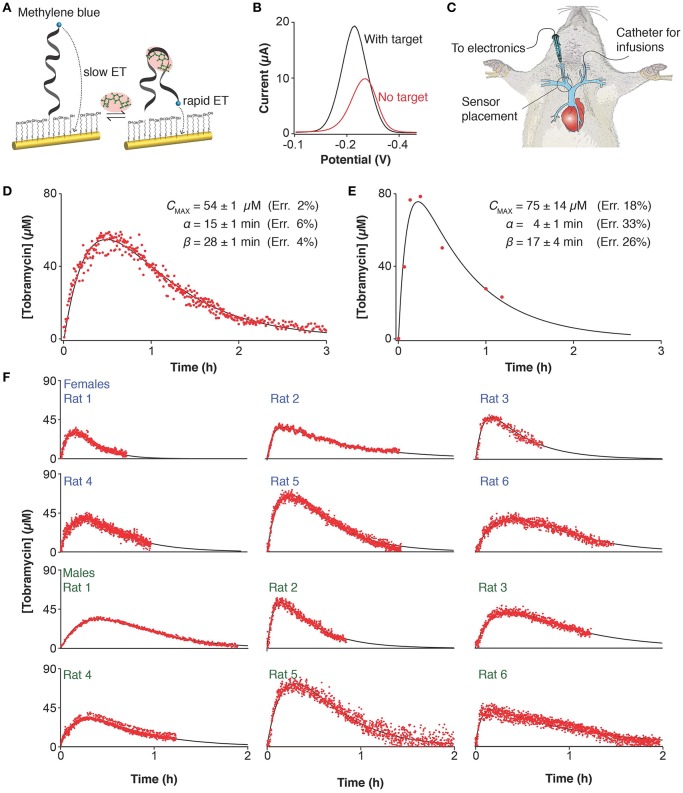
Electrochemical, aptamer-based (E-AB) sensors support the real-time measurement of drug concentrations *in situ* in the body. **(A)** E-AB sensors consist of a redox-reporter-modified aptamer (a nucleic acid selected for its ability to bind the target of interest) tethered to an interrogating electrode. In the presence of the target molecule a binding-induced conformational change alters the rate of electron transfer from the reporter, **(B)** altering the peak currents observed when the sensor is interrogated using square wave voltammetry at 30 and 200 Hz. This electrochemical method is highly sensitive to small changes in electron transfer thus making it ideal for the interrogation of E-AB sensors. **(C)** With a diameter of just 225 μm, the E-AB sensors we employed are narrow enough to emplace inside the jugular vein of live rats, supporting *in-vivo* measurements. The comparison of **(D)** E-AB vs. **(E)** gold standard HPLC-MS pharmacokinetics measured on two independent animals following intramuscular dosing of tobramycin (20 mg/kg) shows that the differences between the two profiles fall well within the range of animal-to-animal variability (see below). **(F)** To highlight animal-to-animal variability we present here pharmacokinetic data collected on 12 individual female and male rats, with weights spanning 350–500 g, illustrating the precision with which the few-second time resolution of E-AB sensors enables the high-precision tracking of the tobramycin's adsorption, distribution, and excretion kinetics.

In response to the pressing need for improved methods of defining patient-specific pharmacokinetics we have developed a platform that can measure drug concentrations *in situ* in the body conveniently and with high frequency, thus enabling unprecedented precision in the determination of pharmacokinetics (Arroyo-Curras et al., 2017b). Our approach employs electrochemical aptamer-based (E-AB) sensors, a platform comprised of a redox-reporter-modified, target-binding aptamer (a nucleic acid selected for its ability to bind the target of interest) that is covalently attached to an interrogating electrode (Figure 1A). This, in turn, undergoes a binding-induced conformational change that alters the approach of the reporter to the electrode surface, producing an easily measurable change in current upon interrogation via square wave voltammetry (Figure 1B). This conformation-change-linked signal transduction mimics the mechanisms employed by naturally occurring receptors in the body (Ricci et al., 2016), rendering the E-AB platform unique among general sensing architectures in its ability to operate *in situ* in the living body (Figure 1C) (Arroyo-Curras et al., 2017a,b). Here, we validate this technology in a rat animal model by comparison to gold-standard *ex-vivo* (HPLC-MS) measurements before demonstrating its unparalleled precision in the determination of drug pharmacokinetics.

## Method

We selected the disposition kinetics of the antibiotic tobramycin as our test bed. This drug is generally dosed following averaged pharmacokinetic data collected from a large population (Inciardi and Batra, 1993) which establishes plasma concentrations below 6 μg/mL (11 μM) as potentially ineffective [depending on the pathogen's minimum inhibitory concentration (Roberts et al., 2008; Cobussen et al., 2015; Baclet et al., 2017)] and above 50 μg/mL (88 μM) as frequently nephrotoxic or ototoxic (Whelton et al., 1976; Moore et al., 1984; Wood et al., 1988; McCormack and Jewesson, 1992; Eliopoulos, 2007; Lee et al., 2017). When coupled with significant patient-to-patient metabolic variability (Avent et al., 2011) the narrowness of this therapeutic window renders tobramycin's dosing a significant clinical challenge (de Velde et al., 2018).

To calibrate our approach against the “gold-standard” analytical method we performed tobramycin E-AB measurements, and blood sampling for HPLC-MS analysis in two independent groups of rats (see Materials and Methods). We did this, as opposed to simultaneously performing E-AB measurements and blood sampling in the same rat, to avoid surgically blocking two major veins of the animals in any single experiment, which would affect the drug's pharmacokinetic profile. Specifically, for the E-AB measurements we emplaced sensors in the right jugular vein of rats and continuously (every 7 s) measured tobramycin levels following IM administration of the drug (20 mg/kg) to the thigh. Our E-AB sensors can measure *in-vivo* plasma tobramycin levels in the range of few (exact limit of detection is 5 μM) to hundreds of micromolar. A full description of the procedure for the surgical placement of E-AB sensors is given in the Materials and Methods section. For HPLC-MS sampling, we collected blood samples (at 0, 1, 5, 10, 30, and 60 min, 1 min collection time) from the left jugular vein of rats, which we then processed to isolate plasma, and stored in ice. Briefly, for the *ex-vivo* analysis of samples via HPLC-MS we first diluted the isolated plasma by 50% in acetonitrile and centrifuged the samples to precipitate out any protein components. We then removed the acetonitrile from the supernatant with dichloromethane, which, after centrifuging, induced a phase partition leaving the tobramycin-containing water at the top, which we subsequently analyzed via HPLC-MS. We calibrated this approach using tobramycin standards and accounted for dilution and matrix effects via standard spiking; we provide a full description in the Materials and Methods section.

## Results

E-AB sensors produced plasma levels and pharmacokinetic parameters closely comparable to those obtained via HPLC-MS measurements (Figures 1D,E). But because E-AB measurements are far more frequent, they produce much more precise pharmacokinetic parameter estimates. Specifically, while the cumbersome nature of blood draws and the limited amount of blood we can remove from a rat without causing undue harm limited our *ex-vivo* measurements to just 7 time points, the 7 s measurement frequency of E-AB measurements leads to many hundreds of data points collected per pharmacokinetic profile (Figures 1D,E). Performing least-squares regression analysis of both the E-AB and HPLC-MS data against a one compartment, open pharmacokinetic model with first order drug absorption and elimination kinetics (Loftsson, 2015) we find that this typically translates into a ~3-fold improvement in the precision with which we measured the uptake phase of the drug, γ, and a ~5-fold improvement in the determination of the elimination phase, ß. Of note, it appears likely that the precision of the later estimate is limited not by the number of data points we collect but instead by the fact that, at this level of precision, ß is no longer a constant as it fluctuates depending on hour-to-hour variations in the animal's kidney function (Arroyo-Currás et al., 2018).

The unprecedented precision of E-AB-derived pharmacokinetic measurements provides an opportunity for new insights into pharmacokinetic variability. To see this, we employed E-AB sensors to record tobramycin pharmacokinetic profiles in a total of 22 Sprague-Dawley rats (12 representative measurements are shown in Figure 1F) following IM administration. To determine the relative variability between subjects, we performed least-squares fit of the data against the one compartment model and performed Monte Carlo propagation of the errors from these fits to determine the 95% confidence interval on each parameter (Table S1). E-AB sensors revealed up to 4-fold differences in the areas under the curve (AUC) recorded between subjects; for example, female rat 1 (19 ± 1 μM h) vs. rat 5 (74 ± 2 μM h), and male rat 11 (24 ± 3 μM h) vs. rat 22 (104 ± 3 μM h) in Table S1.

**Figure 2:**
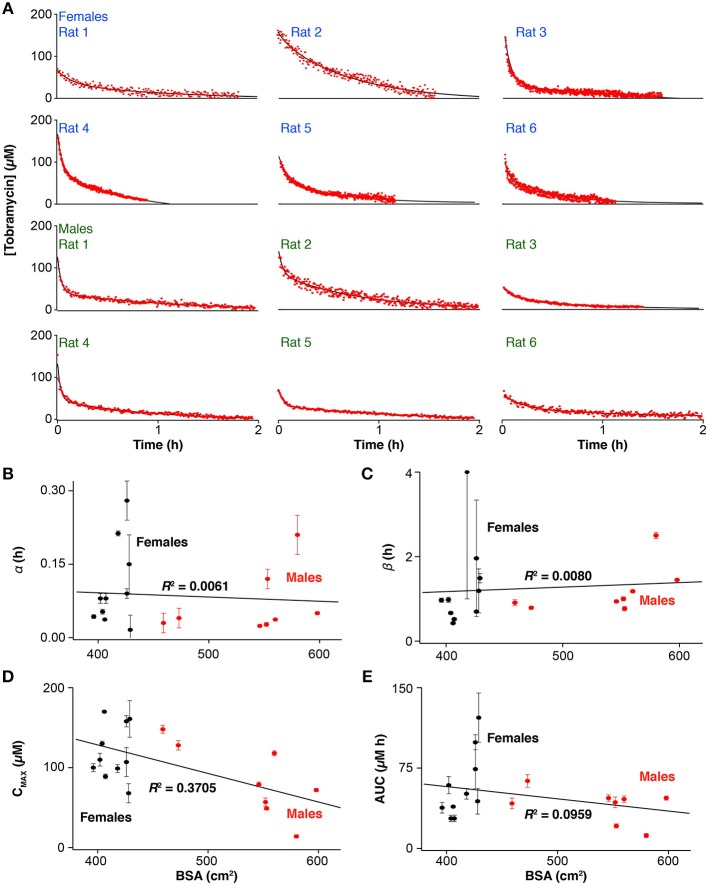
Ultra-high-precision E-AB pharmacokinetic measurements following intravenous administration (20 mg/kg) of tobramycin **(A)** reveal important inter-subject variability in the **(B)** distribution and **(C)** elimination phases, as well as the **(D)** maximum plasma concentration and the **(E)** area-under-the-curve in male, and female Sprague-Dawley rats. Error bars indicate the standard error determined by performing a least-squares fit of the drug profiles to a two-compartment pharmacokinetic model.

In addition to studying tobramycin pharmacokinetics following IM injections, we also explored IV injections, which are the standard of care for patients receiving aminoglycoside therapy (Loewenthal and Dobson, 2010). We did so (over 1-min injection duration) in a total of 17 rats (12 representative measurements are shown in Figure 2A). Given the unprecedented time resolution of E-AB sensors we easily resolve two exponential phases in most of the profiles, corresponding to α, the distribution of the drug and ß, its elimination. Here, again, we calculated pharmacokinetic variability by performing a least-squares fit of each profile, except this time we employed a two compartment open pharmacokinetic model of a bolus injection (Loftsson, 2015). The pharmacokinetic parameters resulting from this analysis (Table S2) demonstrate that although both the distribution and elimination of tobramycin vary significantly between subjects, the major source of variability originates from ß (~6-fold), which is reflected in the area under the drug profiles and thus the absolute dose delivered. Since drug elimination depends strongly on the overall metabolic rate of each subject at the time of measurement, this parameter has a strong influence in the bioavailability of the drug. This observation agrees with a previous report where we demonstrated time-dependent fluctuations in ß within individual animal subjects following serial intravenous injections of tobramycin (Arroyo-Curras et al., 2017b).

The high-precision measurements provided by E-AB sensors highlight the poor precision of existing predictors of tobramycin pharmacokinetics. Specifically, none of the pharmacokinetic parameters extracted from profiles derived from intravenous drug infusions (Figures 2B–E) exhibit any significant correlation to BSA or other body parameter we explored, including weight or sex, the state-of-the-art metrics used in the clinic to determine the dosage of many drugs. As an example, least-squares analysis of the areas under the drug profiles produce a linear correlation coefficient of *R*^2^ = 0.096 (Figure 2E) indicating no significant relation between drug exposure and BSA.

## Discussion

Here we have explored the use of E-AB sensors as a means of easily and rapidly obtaining the pharmacokinetic profiles of individuals with unprecedented precision, using the disposition kinetics of tobramycin in Sprague-Dawley rats as our model system. Comparison with *ex-vivo* measurements performed using gold-standard HPLC-MS highlights both the accuracy of E-AB sensors and the pharmacokinetic precision provided by their far greater temporal resolution. The vastly improved precision of this approach renders it easy to identify significant subject-to-subject variability in drug levels following BSA-adjusted dosing, an approach long considered the gold standard for dosing tobramycin. For example, at the extremes of our animal population receiving intravenous infusions, we observed >50% variance in all pharmacokinetic parameters and no linear correlation between these parameters and BSA (Figures 2B–E) or any of a number of other potential predictors, including weight, and sex. These results confirm the empirically known inaccuracy of BSA, or body-based approaches in general, as metrics for dose determination in this class of drugs (Horrevorts et al., 1985; Touw et al., 1994; Hennig et al., 2008), and, more broadly, emphasize the need for technologies supporting real-time therapeutic drug monitoring of drugs to achieve effective patient-specific, metabolism-responsive drug dosing.

BSA calculations were developed a century ago based on data from healthy adults of average physique which, as demonstrated both by our real-time measurements and empirically by clinicians, are woefully inaccurate (Redlarski et al., 2016). This is particularly true for the most grievously ill patients, who are the very patients for which the margin for clinical error is smallest (Felici et al., 2002). Specifically, clinical studies of the aminoglycosides have reported the need of specialized dosing regimens for obese patients (Bauer et al., 1983). This, in turn, has pushed clinicians to develop modified formulas for specific populations, for example, patients varying by body composition [e.g., obese (Pai, 2012) vs. lean body mass (Lim et al., 2006)] and age [e.g., the elderly (Bevc et al., 2011) vs. children (Vaudry et al., 2009)]. Recent studies have likewise found that BSA-based dosing does not accurately capture drug pharmacokinetics in demographically distinct groups [e.g., Korean (Cho et al., 2010), Japanese (Kouno et al., 2003), and Arab (Al-Khader et al., 2008) patients vs. Caucasian]. Despite the overwhelming evidence that BSA is an inaccurate method for dose determination, however, the U.S. Food and Drug Administration nevertheless continues to recommend BSA-based dose scaling from preclinical studies to human trials (Blanchard and Smoliga, 2015), an approach that many scientists and clinicians discourage (Price and Frazier, 1998). Given the documented variability in patient-specific drug pharmacokinetics and inaccuracy of using BSA-based dosing, our platform helps resolve these issues by providing real-time, individualized drug pharmacokinetics.

More broadly, the ability to measure *in vivo* drug concentrations with seconds resolution offers unprecedented opportunities for pharmacological research and clinical drug therapy. In research, this ability could allow us to study drug pharmacokinetics of large cohorts while strictly controlling disposition variability. In addition, real-time, *in-vivo* drug measurements could also help in the scaling of drug dosages from non-clinical animal studies to clinical human trials, aiming to eliminate the use of ineffective or toxic drug levels during drug development. In the clinic, this platform could be employed for feedback-controlled drug delivery, in which the measured levels of drugs, and disease biomarkers could be used as inputs to achieve health-responsive and personalized dosing drug therapy (Kim and Dionne, 2009; Liu et al., 2014). Thus, the E-AB measurements we presented here illustrate some of these possibilities and will hopefully inspire the development of other platforms supporting continuous, *in-vivo* sensing.

## Materials and Methods

### Chemicals and Materials

Sodium hydroxide, sulfuric acid, tris (hydroxymethyl) aminomethane (Tris), ethylenediaminetetraacetic acid (EDTA), sodium hydrogen phosphate, sodium chloride, potassium chloride, and potassium dihydrogen phosphate were ordered from Fisher Scientific (Waltham, MA). 6-Mercapto-1-hexanol and tris(2-carboxyethyl)phosphine were ordered from Sigma Aldrich (St. Louis, MO). USP grade tobramycin sulfate was purchased from Gold BioTechnology (St. Louis, MO). A 1X stock solution of phosphate buffered saline (PBS) was prepared by mixing 8 g of sodium chloride, 0.2 g of potassium chloride, 1.44 g of sodium hydrogen phosphate, and 0.24 g of potassium hydrogen phosphate in 800 mL of distilled water. The pH of this solution was adjusted to 7.4 using hydrochloric acid and the volume was adjusted to 1 L. A 1X stock solution of Tris-EDTA buffer was prepared by mixing 1 ml of 1 M Tris-HCl (pH 8.0) with 0.2 ml EDTA (0.5 M), adjusting the final volume to 100 mL. All chemicals were used as received.

The E-AB sensors employed here were adapted from previous work (Wang and Rando, 1995; Rowe et al., 2010; Ferguson et al., 2013; Arroyo-Curras et al., 2017b). To fabricate them we ordered methylene-blue-and-thiol-modified DNA constructs from Biosearch Technologies (Novato, CA) with the sequence:

5′ − HS − (CH_2_)_6_ − GGGACTTGGTTTAGGTAATGAGTCCC − (CH_2_)_7_ − NH − MethyleneBlue − 3′.

The 5′ end was modified with a hexanethiol linker and the 3′ end with a carboxy-modified methylene blue attached to the DNA via the formation of an amide bond to a primary amine on a 7-carbon linker. The structure of the exact methylene blue modification is available at the manufacturer's website (Biosearch Technologies, Inc.). The modified DNAs were HPLC purified by the supplier and used as received. Upon receipt each construct was dissolved to 200 μM in 1X Tris-EDTA buffer and frozen at −20°C in individual aliquots until use.

Catheters (22 G) and 1 mL syringes were purchased from Becton Dickinson (Franklin Lakes, NJ). Gold and silver wires (25 μm diameter) were purchased from A-M systems (Sequim, WA). To employ the silver wires as reference electrodes they were immersed in bleach overnight to form a silver chloride film. Heat-shrink polytetrafluoroethylene insulation (PTFE, HS Sub-Lite-Wall, 0.02, 0.005, 0.003 ± 0.001 in, black-opaque, Lot # 17747112-3) to use on the gold and silver wires was purchased from ZEUS (Branchburg Township, CA).

### E-AB Sensor Fabrication

Segments of gold and silver wire 20 cm in length were cut to make sensors. These wires were then insulated, first individually and then together, by applying heat to shrinkable tubing around the body of the wires. The sensor window (i.e., the region without insulation) was approximately 3 mm in length for the gold wire and 6 mm for the silver wire. The edge opposite to the sensor window was left without insulation for a length of 1 cm for both wires. To increase surface area (Arroyo-Curras et al., 2017a) of the as prepared gold electrodes (to obtain larger peak currents) the sensor surface was roughened electrochemically via immersion in 0.5 M sulfuric acid followed by potential jumping between *E*_initial_ = 0.0 V to *E*_high_ = 2.0 V vs. Ag/AgCl, back and forth, for 100,000 pulses. Each pulse was of 2 ms duration with no “quiet time.”

To fabricate sensors an aliquot of the DNA construct was thawed and then reduced for 1 h at room temperature with a 1,000-fold molar excess of tris(2-carboxyethyl)phosphine. A freshly roughened gold electrode was then rinsed in di-ionized water before being immersed in DNA at 200 nM in PBS for 1 h at room temperature. Following this the sensors were immersed overnight at 4°C for 12 h in 20 mM 6-mercapto-1-hexanol in PBS to coat the remaining gold surface and remove non-specifically adsorbed DNA. After this the sensors were rinsed with deionized water and stored in PBS.

### Electrochemical Methods and Data Processing

To determine *in-vivo* drug levels E-AB sensors were interrogated using square wave voltammetry from 0.0 V to −0.5 V vs. Ag/AgCl, using an amplitude of 50 mV, potential step sizes of 1–5 mV, and varying frequencies from 10 to 500 Hz. The files corresponding to each voltammogram were recorded in serial order using macros in CH Instruments software. The post-experiment analysis of results was carried out using a script coded in Igor Pro 7.

### HPLC-MS Validation

The LC-MS/MS analysis was performed on an Agilent 1,290 HPLC coupled with an Agilent 6,470 Triple Quad detector. The column used was an Agilent ZORBAX HILIC Plus 2.1 × 100 mm, 1.8-Micron (p/n: 959758-901). Optimum retention of tobramycin was achieved using an isocratic mobile phase of 90% 20 mM ammonium formate, 0.1% formic acid in water (mobile phase A), 10% Acetonitrile (mobile phase B). The flow rate was 0.2 ml/min and injection volume was 10 ul. The column oven temperature was set to 30°C. Mass spectral setting was in positive ionization mode, with gas temperature at 330°C, gas flow at 13.0 L/min, nebulizer gas at 40 PSI, sheath gas temperature at 395°C, sheath gas flow at 12 L/min, capillary voltage at 2,000 V and nozzle voltage at 0 V. Quantification was performed using multiple reaction monitoring (MRM) of the transitions of m/z 468.3 to m/z 163.1 as quantifier, and m/z 468.3 to m/z 324.1 as qualifier.

Standard stock solutions of tobramycin were prepared in water at a concentration of 1 mg/ml (~1.7 mM). These were diluted to 100 ug/ml (~175 μM) with methanol: water (1:1) as working solution. All solutions were stored in amber bottles and kept in 4°C storage room until use. Matrix matching calibration standards at concentrations of 2,500, 7,500, 15,000, 25,000, 35,000, 50,000 ng/mL were prepared using blank rat serum extract. The limit of detection (LOD) and limit of quantitation (LOQ) were determined by diluting tobramycin standards till concentrations with signal-to-noise close to 3 (LOD) or 10 (LOQ). In this study, LOD for tobramycin is 1 ng/ml and LOQ is 3.3 ng/ml.

To prepare samples for HPLC-MS analysis, 500 uL of rat serum were mixed and vortexed with 500 uL of acetonitrile (ACN) for 3 min, followed by centrifugation at 14.8 × 10^3^ RPMs for 10 min to precipitate out serum proteins. Then 1 ml of supernatant was transferred into a new vial and mixed with 500 uL of dichloromethane (DCM), vortexed for 3 min, followed by centrifugation at 14.8 × 10^3^ for 10 min. In this step, DCM was miscible with ACN and partitioned away from water. Tobramycin stayed in the water phase (the upper layer). 400 uL of this supernatant was mixed and vortexed with 400 uL of deionized water, then recentrifuged at 14.8 × 10^3^ for 10 min to partition out any DCM left. Finally, 600 uL of supernatant was filtered through a 0.22 um Whatman filter and transferred into vials for HPLC-MS analysis.

### *In-vivo* E-AB Measurements

All *in vivo* measurements were performed using a two-electrode setup in which the reference and counter electrodes were a silver wire coated with a silver chloride film as described above. The measurements carried out *in vivo* were recorded using a handheld potentiostat (Model 1242 B) from CH Instruments (Austin, TX). A 30 min sensor baseline was established before the first drug infusion. For IV injections, a 3 mL syringe filled with tobramycin solution was connected to the sensor-free catheter (placed in the jugular opposite that in which the sensor was emplaced) and to a motorized syringe pump (KDS 200, KD Scientific Inc., Holliston, MA). After establishing a stable baseline, the drug was infused through this catheter at a rate of 0.25 mL/min, using a stock solution of tobramycin (0.1 M solution; homemade). During and after drug infusion, recordings were taken every 7 s for up to 3 h. The real-time plotting and analysis of voltammetric data were carried out with the help of custom MATLAB scripts.

### Regression Analysis Using Monte Carlo Propagation

We performed non-linear regression analysis of our concentration/time data using two pharmacokinetic models: (1) a two-compartment open model for intravenous injections and (2) a one-compartment open model with first-order drug absorption for intramuscular injections (Loftsson, 2015). The equations employed in the regressions were the following:

For IV injections:

(1) $$\begin{array}{r} C_{P}=Ae^{-\frac{t}{\alpha }}+Be^{-\frac{t}{\beta }} \end{array}$$

For IM injections:

(2) $$\begin{array}{r} C_{P}=\frac{C_{MAX}}{\gamma \left(\frac{1}{\gamma }-\frac{1}{\beta }\right)}\left[e^{-\frac{t}{\beta }}-e^{-\frac{t}{\gamma }}\right] \end{array}$$

We carried out our data analysis using in-house-coded Matlab® scripts. Our analysis consisted of mathematically fitting the pharmacokinetic profile corresponding to each drug injection to either Equation 1 or Equation 2, depending on the route of administration. During the regression analysis, the best fit of the corresponding model to the experimental data was determined by minimizing the least-square errors via Nelder-Mead simplex algorithm (Lagarias et al., 1998), in which the parameters were allowed to float unconstrained to obtain the values of: the maximum drug concentration, *C*_MAX_, and the drug's lifetimes for the distribution, α, elimination, β, and uptake, γ, phases. We note that α specifically refers to distribution of the drug from the bloodstream into tissues following an IV injection and γ is the uptake phase following an IM injection, i.e., the diffusion of the drug from muscle tissue and into the bloodstream. We performed Monte Carlo iterations to provide the variability distribution of the calculated parameters and the area under the curve. Specifically, in each Monte Carlo iteration we propagated the root-mean-square error into our experimental data and fit the resulting dataset to the model to extract a new set of pharmacokinetic parameters. We repeated this process for 5,000 times (a number of iterations large enough to ensure convergence of the simulation) for each injection dataset and then used the newly generated pool of parameters to report the uncertainty on the pharmacokinetic parameters as the 95% confidence interval derived from the distribution of parameters.

### Animals

Healthy adult (mean age = 9 weeks) male (mean weight = 300 g) and female (mean weight = 200 g) Sprague-Dawley rats were ordered from Charles River (Hollister, CA) and same-sex pair-housed with autoclaved wood chip bedding in a 12 h light/dark cycle (all procedures were conducted during the middle half of the light phase of the light/dark cycle), temperature (mean = 25° C), and humidity (mean = 71%) controlled vivarium with *ad libitum* access to food and water. All animals were allowed a minimum of 48 h to habituate to the vivarium prior to the experiment and assessed for welfare prior to use. All animals were naïve to the drug and test procedures. Selection of our animal cohorts initially started with a group of male rats (*n* = 8) for intramuscular (IM) and, upon noting substantial inter-subject variability, we were motivated to repeat the IM injections in males and add females to investigate potential variability in pharmacokinetics following IV injections between the two sexes. This led to a discrepancy in the number of animals in each sex for the intramuscular injection condition. Except for the initial group of males, rats of each sex were randomly allocated to either the IM or IV group. A total of 40 animals were used for all procedures, 22 for IM procedures (15 male and 7 female) and 18 for IV procedures (8 male and 10 female). All of the experimental procedures were approved by the Institutional Animal Care and Use Committee (IACUC) of the University of California Santa Barbara and adhered to the guidelines given by the NIH Guide for Care and Use of Laboratory Animals (2011).

### Study Design

Single animals serve as the experimental units for this study and analyses were done on an individual basis. Overall, 4 separate groups of animals were analyzed: male and female rats injected either with intramuscular (male *n* = 15, female *n* = 7) or intravenous (male *n* = 8, female *n* = 10) drug. Following surgical preparation, baseline recordings were initially taken to serve as a control comparison for post-injection recordings. Analyses primarily focused on comparing subject-to-subject differences in drug pharmacokinetics.

### Surgical Procedures

For *in vivo* measurements rats were induced under 5% isoflurane anesthesia in a Plexiglas anesthesia chamber and then maintained on 2–3% isoflurane gas via a nosecone for the duration of the experiment. While anesthetized, E-AB sensors were inserted into the right jugular vein and for rats receiving IV tobramycin, an infusion line was inserted into the opposite, left, jugular vein. Briefly, the area above each jugular vein was shaved and cleaned with betadine, and 70% ethanol. A small incision was made above each vein, and then each vein was isolated. A small hole was cut into each vein with spring-loaded microscissors. Into one, we inserted the E-AB sensor and in the other, we inserted a silastic catheter constructed with a bent steel cannula with a screw-type connector (Plastics One, Roanoke, VA) and silastic tubing (11 cm, i.d., 0.64 mm, o.d., 1.19 mm, Dow Corning, Midland, MI) for infusions. Both the E-AB sensor and the infusion line were tied into place with sterile 6–0 silk suture (Fine Science Tools, Foster City, CA), then 30 units of heparin was infused into the vein prior to establishing baseline recordings, and response to drug challenge (see above). Following experimental procedures, all animals were immediately euthanized.

A separate group of rats (*n* = 2) were used for *ex vivo* measurements of tobramycin. Rats were anesthetized as above and an infusion line was emplaced in the left jugular and a collection line (same as infusion line) was emplaced in the right jugular. Then, rats received an IM injection of tobramycin (20 mg/kg) and blood was periodically collected by applying negative pressure to a syringe attached to the infusion line. Blood was centrifuged at 2,500 RPM to separate serum, serum was extracted with a micropipette, and then stored for subsequent analyses by HPLC-MS.

### Experimental Outcomes

The primary goal of these experiments was to study subject-to-subject variability in pharmacokinetic measurements. This was assessed by comparing E-AB measurements of *in-vivo* drug levels across animals of differing sex and BSA following either IM or IV infusion of the target compound (tobramycin).

## Data Availability

The datasets generated for this study are available on request to the corresponding author.

## Author Contributions

PV, CS, NA-C, KP, and TK participated in the research design. PV, CS, NA-C, and WL conducted the experiments. PV, CS, NA-C, GO, and AK performed the data analysis. PV, CS, NA-C, GO, KP, and TK contributed to the manuscript editing.

### Conflict of Interest Statement

NA-C and KP hold a number of patents in the area of E-AB sensors and KP serves on the scientific advisory boards of and maintains a small amount of ownership in two companies that are attempting to commercialize E-AB sensors. The remaining authors declare that the research was conducted in the absence of any commercial or financial relationships that could be construed as a potential conflict of interest.
