# Supplementary material for: Ultra-High-Precision, in-vivo Pharmacokinetic Measurements Highlight the Need for and a Route Toward More Highly Personalized Medicine
Source: Front Mol Biosci. 2019 Aug 16;6:69. doi: 10.3389/fmolb.2019.00069 (PMC6707041; doi:10.3389/fmolb.2019.00069)
Supplement: Supplementary file 1 [file Table_1.docx]

**Table S1.** Pharmacokinetic parameters obtained by regression analysis of intramuscular (IM) injections.

| **Females** |  |  |  |  |  |  |
| --- | --- | --- | --- | --- | --- | --- |
| **ID** | **BSA^b^**  **(cm^2^)** | **Dose**  **(mg/kg)** | **C_MAX_ (µM)^a^** | **γ (h) ^a^** | ***β* (h) ^a^** | **AUC**  **(µM h) ^a^** |
| Rat 1 | 443 | 80 | 30±7 | 0.18±0.02 | 0.29±0.03 | 19±1 |
| Rat 2 | 453 | 80 | 38±1 | 0.100±0.002 | 1.09±0.01 | 47±1 |
| Rat 3 | 391 | 81 | 49±2 | 0.13±0.01 | 0.62±0.04 | 44±5 |
| Rat 4 | 429 | 81 | 40±2 | 0.23±0.01 | 0.64±0.02 | 43±2 |
| Rat 5 | 436 | 81 | 65±2 | 0.23±0.01 | 0.66±0.01 | 74±2 |
| Rat 6 | 445 | 82 | 43±1 | 0.550±0.003 | 0.650±0.003 | 65±5 |
| Rat 7 | 412 | 84 | 53±2 | 0.09±0.01 | 0.49±0.02 | 39±4 |
| **Males** |  |  |  |  |  |  |
| Rat 8 | 497 | 40 | 36±1 | 0.7±0.1 | 0.6±0.1 | 53±1 |
| Rat 9 | 482 | 59 | 40±5 | 0.31±0.03 | 1.16±0.14 | 68±9 |
| Rat 10 | 494 | 60 | 78±10 | 0.16±0.05 | 0.47±0.13 | 63±21 |
| Rat 11 | 509 | 61 | 40±2 | 0.050±0.004 | 0.46±0.02 | 24±3 |
| Rat 12 | 553 | 62 | 40±1 | 0.31±0.01 | 1.07±0.03 | 65±2 |
| Rat 13 | 503 | 62 | 68±1 | 0.120±0.003 | 0.58±0.01 | 59±2 |
| Rat 14 | 542 | 80 | 76±10 | 0.33±0.04 | 0.41±0.04 | 77±2 |
| Rat 15 | 477 | 80 | 62±6 | 0.43±0.01 | 0.43±0.01 | 72±4 |
| Rat 16 | 484 | 80 | 70±4 | 0.32±0.01 | 0.68±0.03 | 91±3 |
| Rat17 | 529 | 80 | 32±1 | 0.25±0.01 | 0.88±0.02 | 43±1 |
| Rat 18 | 527 | 81 | 52±1 | 0.120±0.004 | 0.57±0.01 | 43±2 |
| Rat 19 | 512 | 81 | 40±1 | 0.10±0.01 | 1.51±0.02 | 64±2 |
| Rat 20 | 525 | 81 | 62±2 | 0.10±0.01 | 1.27±0.04 | 88±15 |
| Rat 21 | 529 | 82 | 37±5 | 0.6±0.1 | 0.7±0.1 | 58±1 |
| Rat 22 | 477 | 87 | 76±2 | 0.28±0.01 | 0.77±0.02 | 104±3 |

^a^ Errors correspond to 95% confidence intervals determined using Student’s t-test.

^b^ BSA values computed using the equation: BSA = 9.83x(Weight)^2/3^, taken from (Gouma et al., 2012).

**Table S2.** Pharmacokinetic parameters obtained by regression analysis of intravenous injections.

| **Females** |  |  |  |  |  |  |
| --- | --- | --- | --- | --- | --- | --- |
| **ID** | **BSA**  **(cm^2^)^b^** | **Dose**  **(mg/kg)** | **C_MAX_**  **(µM)^a^** | **α (h) ^a^** | ***β* (h) ^a^** | **AUC**  **(µM h) ^a^** |
| Rat 23 | 426 | 20 | 107±2 | 0.28±0.04 | 1.96±1.38 | 74±18 |
| Rat 24 | 426 | 20 | 158±4 | 0.09±0.01 | 0.70±0.02 | 99±7 |
| Rat 25 | 428 | 20 | 68±2 | 0.15±0.06 | 1.19±0.52 | 44±12 |
| Rat 26 | 429 | 20 | 161±9 | 0.016±0.003 | 1.49±0.11 | 122±23 |
| Rat 27 | 404 | 20 | 130±5 | 0.053±0.005 | 0.667±0.03 | 28±3 |
| Rat 28 | 418 | 20 | 99±1 | 0.213±0.005 | 4±3 | 51±5 |
| Rat 29 | 406 | 20 | 170±2 | 0.037±0.001 | 0.425±0.004 | 39±1 |
| Rat 30 | 407 | 20 | 89±2 | 0.08±0.01 | 0.52±0.03 | 28±3 |
| Rat 31 | 402 | 20 | 110±6 | 0.08±0.01 | 0.98±0.06 | 59±8 |
| Rat 32 | 396 | 20 | 100±4 | 0.043±0.004 | 0.97±0.05 | 38±5 |
| **Males** |  |  |  |  |  |  |
| Rat 33 | 560 | 20 | 118±2 | 0.037±0.002 | 1.18±0.03 | 46±3 |
| Rat 34 | 473 | 20 | 128±12 | 0.04±0.02 | 0.79±0.03 | 63±6 |
| Rat 35 | 459 | 20 | 148±74 | 0.03±0.02 | 0.91±0.07 | 42±5 |
| Rat 36 | 553 | 20 | 49±1 | 0.12±0.02 | 0.77±0.05 | 21±2 |
| Rat 37 | 580 | 20 | 50±1 | 0.21±0.04 | 2.5±0.7 | 12±2 |
| Rat 38 | 552 | 20 | 57±1 | 0.027±0.004 | 1.00±0.05 | 43±5 |
| Rat 39 | 598 | 20 | 72±2 | 0.050±0.003 | 1.45±0.02 | 47±2 |
| Rat 40 | 546 | 20 | 79±2 | 0.024±0.003 | 0.94±0.03 | 47±3 |

^a^ Errors correspond to 95% confidence intervals determined using Student’s t-test.

^b^ BSA values computed using the equation: BSA = 9.83x(Weight)^2/3^, taken from (Gouma et al., 2012).
